# NLRP3 Inflammasome: A New Pharmacological Target for Reducing Testicular Damage Associated with Varicocele

**DOI:** 10.3390/ijms22031319

**Published:** 2021-01-28

**Authors:** Pietro Antonuccio, Antonio Girolamo Micali, Carmelo Romeo, Jose Freni, Giovanna Vermiglio, Domenico Puzzolo, Francesco Squadrito, Natasha Irrera, Herbert R. Marini, Rosa Alba Rana, Giovanni Pallio, Letteria Minutoli

**Affiliations:** 1 Department of Human Pathology of Adult and Childhood “Gaetano Barresi”, University of Messina, 98125 Messina, Italy; pantonuccio@unime.it (P.A.); romeoc@unime.it (C.R.); 2 Department of Biomedical, Dental, Morphological and Functional Imaging Sciences, University of Messina, 98125 Messina, Italy; amicali@unime.it (A.G.M.); freni.jose.89@gmail.com (J.F.); giovanna.vermiglio1@unime.it (G.V.); puzzolo@unime.it (D.P.); 3 Department of Clinical and Experimental Medicine, University of Messina, 98125 Messina, Italy; fsquadrito@unime.it (F.S.); nirrera@unime.it (N.I.); hrmarini@unime.it (H.R.M.); lminutoli@unime.it (L.M.); 4 Department of Medicine and Aging Sciences, University G. d’Annunzio, Chieti and Pescara, 66100 Chieti, Italy; r.rana@unich.it

**Keywords:** varicocele, testis, NLRP3 inflammasome, A_2A_ receptor, PDRN, selenium, nutraceuticals

## Abstract

Many bioactive natural compounds are being increasingly used for therapeutics and nutraceutical applications to counteract male infertility, particularly varicocele. The roles of selenium and Polydeoxyribonucleotide (PDRN) were investigated in an experimental model of varicocele, with particular regard to the role of NLRP3 inflammasome. Male rats underwent sham operation and were daily administered with vehicle, seleno-L-methionine (Se), PDRN, and with the association Se-PDRN. Another group of rats were operated for varicocele. After twenty-eight days, sham and varicocele rats were sacrificed and both testes were weighted and analyzed. All the other rats were challenged for one month with the same compounds. In varicocele animals, lower testosterone levels, testes weight, NLRP3 inflammasome, IL-1β and caspase-1 increased gene expression were demonstrated. TUNEL assay showed an increased number of apoptotic cells. Structural and ultrastructural damage to testes was also shown. PDRN alone significantly improved all considered parameters more than Se. The Se-PDRN association significantly improved all morphological parameters, significantly increased testosterone levels, and reduced NLRP3 inflammasome, caspase-1 and IL-1β expression and TUNEL-positive cell numbers. Our results suggest that NLRP3 inflammasome can be considered an interesting target in varicocele and that Se-PDRN may be a new medical approach in support to surgery.

## 1. Introduction

Varicocele is a testicular pathology occurring as a consequence of abnormal dilation and/or tortuosity of the veins of the pampiniform plexus [1]. It is considered a relevant clinical problem all over the world, as it is responsible for infertility in men [2].

The exact pathophysiological mechanisms relating varicocele and consequent infertility are still unidentified; however, scrotal hyperthermia, microcirculation disturbance of the testis, hypoxia and oxidative stress appear to play important roles [3]. In particular, reactive oxygen species (ROS) generation seems to play a crucial role in the detrimental cascade induced by varicocele [4,5]. Even if small amounts of ROS are required for capacitation, acrosome reaction and consequent fertilization [6], men with varicocele have significantly increased ROS in addition to elevated DNA fragmentation, causing poor sperm quality. Under these conditions, ROS can stimulate an amplified inflammatory response characterized by increased cytokine expression, and promote apoptosis [7].

Furthermore, it was demonstrated that ROS might play a prominent role in NLR pyrin domain containing 3 (NLRP3) inflammasome activation, which inhibited the blocking of ROS with chemical scavengers [8]. Inflammasomes are multimolecular complexes assembled intracellularly in response to various activators [8]. The NLRP3 inflammasome is, at present, the best-described inflammasome and is made by the NOD-like receptor NLRP3, by the adapter protein “apoptosis-associated speck-like protein containing a caspase recruitment domain” (ASC) and by the procaspase-1. When NLRP3 inflammasome is activated, procaspase-1 cleaves and forms the active caspase-1 [9,10], leading to the secretion of interleukin (IL)-1β and IL-18 [8] and to a rapid inflammatory form of cell death called “pyroptosis” [11]. This is characterized by plasma membrane rupture, cytoplasmic swelling, osmotic lysis, DNA cleavage and the release of further pro-inflammatory cellular contents. Recently, in an experimental model of varicocele, an upregulation of the levels of NLRP3 inflammasome was demonstrated [12], which was decreased by the administration of resveratrol, a nutraceutical compound provided of anti-inflammatory properties. Furthermore, the presence of the NLRP3 inflammasome components in the semen of varicocele patients was also shown [13].

Even if varicocele is considered surgically correctable and the benefit of varicocelectomy has been demonstrated [1,14], not all men showed positive effects in terms of fertility potential [15], particularly those with subclinical varicocele [16]. For this reason, several therapeutic medical strategies have been proposed as a support to surgery in order to improve testicular structure with positive effects on varicocele-induced infertility [17,18,19,20]. Anti-oxidant use is considered, among the proposed ideas, to be the most appropriate therapeutic approach to reduce the effects of varicocele [21,22,23].

Selenium (Se) is an essential trace element with anti-inflammatory and anti-oxidative properties [24,25], which is also active in detoxification from heavy metals, such as cadmium and lead [26,27]. In fact, Se, in the testes of chicken treated with Pb, was able to moderate the pro-inflammatory cytokines cascade through the reduction in the ROS/NF-kB/NLRP3/caspase-1/IL-1β signaling pathway [28].

Regarding the varicocele-induced testicular damage, the treatment with sodium selenite was able to increase the activities of antioxidant enzymes, such as CAT, SOD, and GPX, and reduce lipid peroxidation [29]. In humans, combined treatment with antioxidants (vitamin E-selenium-folic acid) after varicocelectomy improved sperm parameters by removing ROS from the environment [30].

Polydeoxyribonucleotide (PDRN) is the active fraction extracted from trout spermatozoa [31] and, through stimulation of adenosine A_2A_ receptor (ADORA2A), can contrast several harmful mechanisms observed in pathological conditions of heavy metal challenge [32] or low tissue perfusion, such as varicocele [17,18,33]. In this experimental model, our group showed that PDRN restored spermatogenic function, improving neoangiogenesis [17,18] and modulating the inhibitors of apoptosis proteins (IAPs), such as neuronal apoptosis inhibitory protein (NAIP) and survivin [33].

Based on this background, considering (i) that no morphological analyses have been conducted on the apoptosis and on the immunoreactivity of caspase-1 in varicocele testes, in previous studies, (ii) the lack of data regarding the effects of the association Se plus PDRN on varicocele-induced testicular damage, and (iii) that many bioactive natural compounds are increasingly used for therapeutics and nutraceutical applications to counteract male infertility, we aimed to demonstrate the protective role of the association Se plus PDRN on the seminiferous epithelium in rats experimentally exposed to varicocele, with particular regard to their role of NLRP3 inflammasome expression, looking at its mechanism of action, and aiming to understand the effects of this new medical approach on the structural organization of the testis.

## 2. Results

### 2.1. Effects of Se, PDRN and Their Association on Testis Weight

The weight of both testes of all the examined groups was shown in Table 1. No significant differences were observed in all these parameters among sham groups: therefore, for a better comprehension, only one value is indicated for sham. Varicocele rats showed an operated and contralateral testes weight significantly lower than sham, even if contralateral testes weight was significantly greater than operated. In varicocele rats treated with Se, both operated and contralateral testes weight was significantly lower than sham rats. The testes of varicocele rats treated with PDRN were still significantly lower than sham, while contralateral testes of the same group were closer to sham weight. Instead, in varicocele rats treated with Se plus PDRN, the weight of both operated and contralateral testes was improved and close to sham.

### 2.2. Effects of Se, PDRN and Their Association on Testosterone Levels

The levels of testosterone in all the examined groups were shown in Table 1. Testosterone levels were similar among sham groups: therefore, for a better comprehension, only one value is indicated for sham. Se administration produced an increase in testosterone levels (+30%), even if this was significantly lower than in sham groups. PDRN induced a significant increase in testosterone (+49%), when compared to varicocele group. However, in varicocele rats treated with Se plus PDRN, testosterone levels were significantly higher (+56%) and close to sham groups.

### 2.3. Effects of Se, PDRN and Their Association on Glutathione (GSH) and Glutathione Peroxidase (GPx)

A significant decrease in GSH content and in GPx activity were observed in varicocele rats (*p* < 0.05 vs. sham). When varicocele rats were treated with Se or PDRN, both GSH content and GPx activity showed a significant increase compared to varicocele group (*p* < 0.05 vs. varicocele rats). The association Se plus PDRN markedly increase GSH content and GPx activity, demonstrating a greater effect than that of the two compounds alone (*p* < 0.0001 versus varicocele rats) Table 2.

### 2.4. Effects of Se, PDRN and Their Association on NLRP3, IL-1β and Caspase-1 Expression

**Figure 1:**
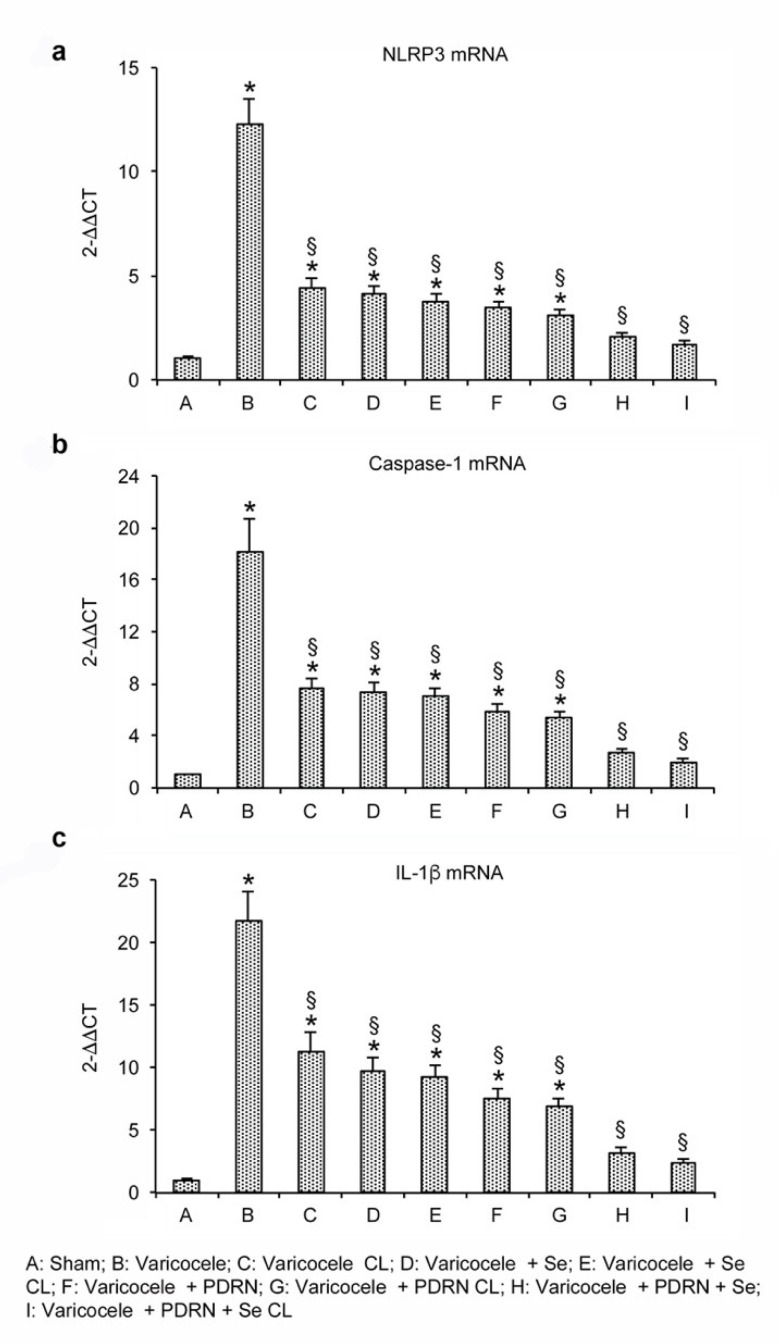
Real-time PCR analysis for NLRP3 (**a**), caspase-1 (**b**) and IL-1β (**c**) in the testes from rats of sham, varicocele, varicocele contralateral, varicocele plus Se (0.4 mg/kg/day i.p.), varicocele plus Se contralateral, varicocele plus PDRN (8 mg/kg/day i.p.), varicocele contralateral plus PDRN, varicocele plus Se plus PDRN, varicocele contralateral plus Se plus PDRN groups. * = *p* < 0.05 versus sham; § = *p* < 0.05 versus varicocele. Bars represent the mean ± SE of 7 experiments.

**Figure 2:**
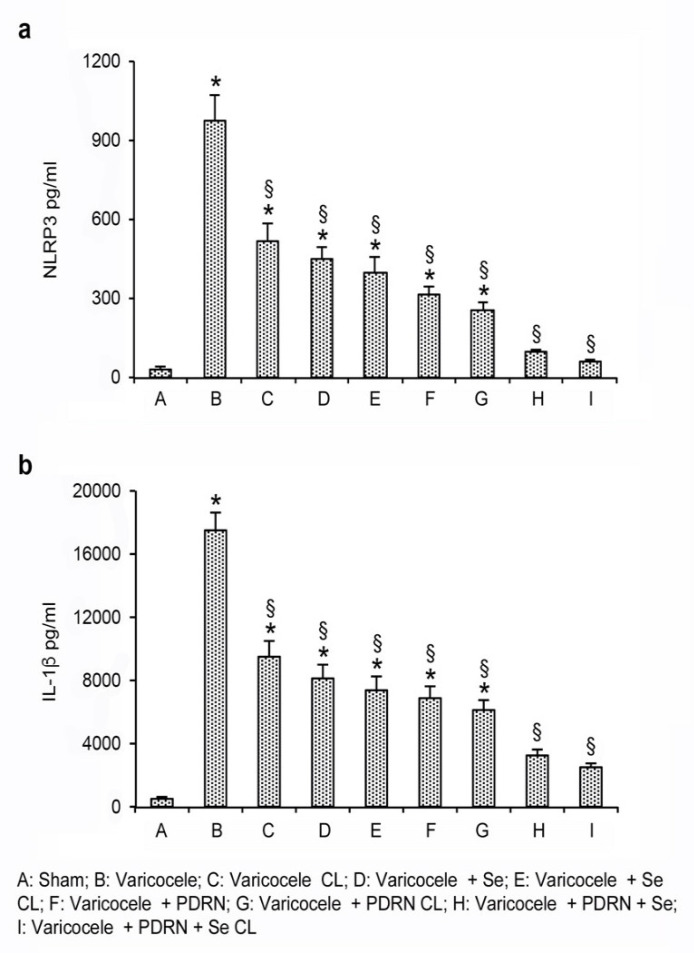
Quantitative evaluation of NLRP3 (**a**) and IL-1β (**b**) levels in the testes from rats of sham, varicocele, varicocele contralateral, varicocele plus Se (0.4 mg/kg/day i.p.), varicocele plus Se contralateral, varicocele plus PDRN (8 mg/kg/day i.p.), varicocele contralateral plus PDRN, varicocele plus Se plus PDRN, varicocele contralateral plus Se plus PDRN groups. * = *p* < 0.05 versus sham; § = *p* < 0.05 versus varicocele. Bars represent the mean ± SE of 7 experiments.

Varicocele animals showed increased mRNA expression of the NLRP3 inflammasome when compared to sham and also to contralateral testes. The treatment with Se or PDRN significantly reduced NLRP3 mRNA expression in both operated and contralateral testes when compared to the testes of varicocele rats (*p* < 0.0001 versus varicocele rats; Figure 1a). The association of Se plus PDRN markedly reverted the increase in the message of NLRP3, demonstrating a greater effect than that of the two compounds alone (*p* < 0.0001 versus varicocele rats; *p* < 0.0001 versus varicocele + Se; *p* < 0.0001 versus varicocele + PDRN; Figure 1a). Overlapping results were observed when mature proteins were used as readouts (Figure 2a).

To explore the activation of the inflammatory cascade triggered by NLRP3, we evaluated the downstream signals, and, more specifically, the expression of caspase-1 and IL-1β mRNAs in the testes of the same groups of animals. Varicocele rats showed an increased expression of the downstream products of the NLRP3 (*p* < 0.0001 versus sham operated; Figure 1b,c). On the contrary, both Se and PDRN treatment reduced caspase-1 and IL-1β levels in testes of varicocele animals (*p* < 0.0001 versus varicocele; Figure 1b,c). The association Se and PDRN more efficiently blunted the increase in caspase-1 and IL-1β expression, thus demonstrating a greater effect than Se and PDRN alone in reducing the downstream products of the NLRP3 (*p* < 0.0001 versus varicocele alone; *p* < 0.0001 versus varicocele + Se; *p* < 0.0001 versus varicocele + PDRN; Figure 1b,c). Similar results were observed when the levels of IL-1β mature proteins were investigated (Figure 2b).

### 2.5. Administration of Se, PDRN and Their Association Counteracts Testes Changes

**Figure 3:**
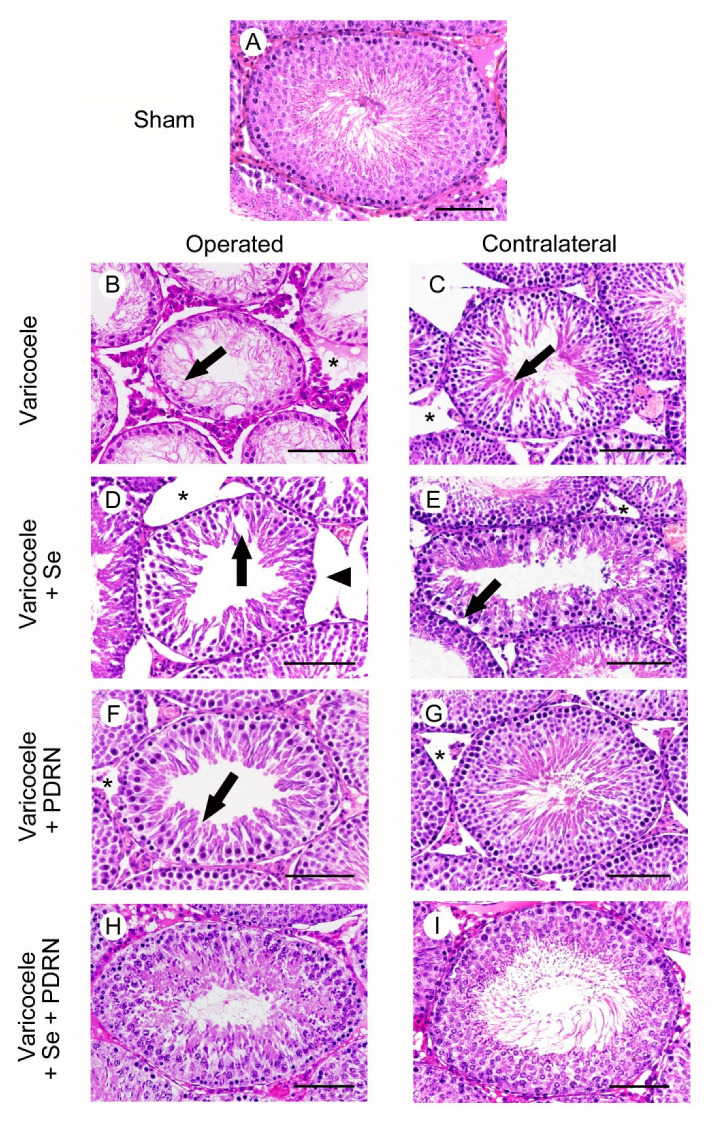
Structural organization of testes from rats of sham, varicocele, varicocele contralateral, varicocele plus Se (0.4 mg/kg/day i.p.), varicocele plus Se contralateral, varicocele plus PDRN (8 mg/kg/day i.p.), varicocele contralateral plus PDRN, varicocele plus Se plus PDRN, varicocele contralateral plus Se plus PDRN groups. (**A**): In sham testes, the seminiferous tubules have normal morphology. (**B**): Varicocele rats. The seminiferous epithelium shows reduced thickness with spermatogonia arranged on 1–2 rows and residual condensed sperm tails (arrow). In the extratubular compartment, a marked edema is evident (*). (**C**): Contralateral testes of the same group. The seminiferous tubules show many elongated spermatids (arrow). The extratubular compartment is edematous and hyperemic (*). (**D**): In varicocele operated rats treated with Se, the seminiferous epithelium is detached from the basement membrane (arrowhead) and many large clefts are present in its wall (arrow). The extratubular compartment is enlarged (*). (**E**): In the contralateral testes of the same group, peripheral spermatogonia are often detached from the basement membrane (arrow). The extratubular compartment shows hyperemic blood vessels and edema (*). (**F**): Varicocele operated rats treated with PDRN. The seminiferous epithelium shows intercellular clefts among round or elongated spermatids (arrow). A mild edema is present in the extratubular compartment (*). (**G**): Contralateral testes of the same group. The seminiferous epithelium has many elongated spermatids and mature spermatozoa; the extratubular compartment shows only a mild edema (*). (**H**,**I**): In the seminiferous tubules of both operated and contralateral testes of varicocele rats treated with Se plus PDRN, the seminiferous epithelium is close to normal. (Scale bar = 50 µm).

All animals of sham groups showed the same morphology after HE stain. As a consequence, for clarity of results, only one image is provided as a representative of sham (Figure 3A) and a single datum is provided for MTD and JS (Table 1). In the testes of sham rats, the normal morphology of the seminiferous tubules was evident (Figure 3A). In the testes of varicocele rats (Figure 3B; Table 1), the seminiferous tubules showed a sharp reduction in their MTD and an epithelium that was highly reduced in thickness, with spermatogonia arranged on 1-2 rows and residual condensed sperm tails: JS was significantly low. The extratubular compartment was enlarged, owing to the presence of a marked edema. In contralateral testes of the same group (Figure 3C; Table 1), the seminiferous tubules diameter was significantly reduced and many spermatids and some immature spermatozoa were present. The extratubular compartment was edematous and hyperemic. In varicocele rats treated with Se (Figure 3D; Table 1), the MTD was higher but the seminiferous epithelium was often detached from the basement membrane and many large clefts were present in its wall, as indicated by the low JS. The extratubular compartment was enlarged, owing to an evident edema. In the contralateral testes of the same group (Figure 3E; Table 1), elongated spermatids were present in the tubular wall (significantly higher MTD and JS), but peripheral spermatogonia were often detached from the basement membrane. The extratubular compartment showed hyperemic blood vessels and interstitial edema. The testes of varicocele rats treated with PDRN (Figure 3F; Table 1) showed larger seminiferous tubules with intercellular clefts among round or elongated spermatids; JS was still significantly low. A mild edema was present in the extratubular compartment. In contralateral testes of the same group (Figure 3G; Table 1), seminiferous tubules with close to normal MTD, many elongated spermatids and mature spermatozoa and a significantly higher JS were demonstrated; a mild edema enlarged the extratubular compartment. In the seminiferous tubules of both operated and contralateral testes of varicocele rats treated with Se plus PDRN (Figure 3H,I; Table 1), the seminiferous epithelium had MTD, structural organization and JS close to sham.

### 2.6. Administration of Se, PDRN and Their Association Modulates Sperm Cells Apoptosis

**Figure 4:**
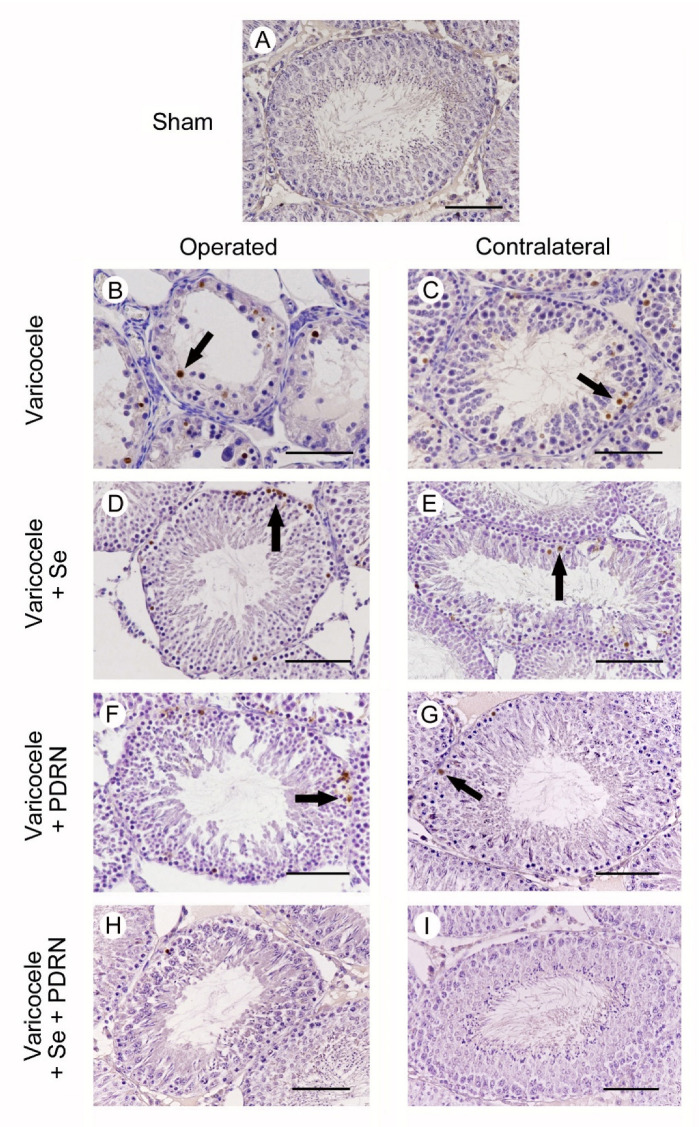
Assessment of apoptosis in the testes with TUNEL staining technique from rats of sham, varicocele, varicocele contralateral, varicocele plus Se (0.4 mg/kg/day i.p.), varicocele plus Se contralateral, varicocele plus PDRN (8 mg/kg/day i.p.), varicocele contralateral plus PDRN, varicocele plus Se plus PDRN, varicocele contralateral plus Se plus PDRN groups. (**A**): In sham rats, no TUNEL positive cells can be observed. (**B**): In the testes of varicocele rats, a large number of TUNEL-positive germ cells (arrow), placed along the wall of the tubules, are observed. (**C**): In the contralateral testes of the same rats, some isolated TUNEL-positive germ cells are present in the peripheral part of the seminiferous tubules (arrow). (**D**): In varicocele rats treated with Se, many TUNEL-positive cells are present in the periphery of the seminiferous tubules (arrow). (**E**): In the contralateral testes of the same group, some TUNEL-positive cells are located in the external part of the tubules (arrow). (**F**): In the testes of varicocele rats treated with PDRN, the number of TUNEL-positive cells is reduced, and they are located along the peripheral part of the tubules (arrow). (**G**): In contralateral testes of the same group, only a few TUNEL-positive cells (arrow) are present in the seminiferous epithelium. H, I: In the seminiferous tubules of both operated and contralateral testes of varicocele rats treated with Se plus PDRN, very rare or no TUNEL-positive cells are observed. (Scale bar: 50 µm).

All animals of sham groups showed the same morphology after TUNEL assay. As a consequence, for the clarity of results, only one image is provided as representative of sham (Figure 4A) and a single datum is provided for TWAC and apoptotic index (Table 3). No TUNEL-positive cells were detected in the seminiferous tubules of sham animals. On the contrary, in the testes of varicocele rats, a large number of TUNEL-positive germ cells, placed along the wall of the tubules, were observed (Figure 4B). In fact, both TWAC and apoptotic index were significantly higher than those observed in the sham group (Table 3). By contrast, in the contralateral testes of the same rats, some isolated TUNEL-positive germ cells were present in the peripheral part of the seminiferous tubules (Figure 4C). Both TWAC and apoptotic index were significantly lower than those of varicocele rats (Table 3). In varicocele rats treated with Se, many TUNEL-positive cells were present in the periphery of the seminiferous tubules; TWAC and apoptotic index were significantly reduced (2.5-fold and 1.4-fold, respectively) (Figure 4D). In the contralateral testes of the same group, some TUNEL-positive cells were demonstrated in the external part of the tubules. TWAC was significantly reduced (Figure 4E; Table 3). In the testes of varicocele rats treated with PDRN, the number of TUNEL-positive cells was reduced, as was also indicated by TWAC and apoptotic index values (Table 3), and they were located along the peripheral part of the tubules (Figure 4F). In the contralateral testes of the same group, only a few TUNEL-positive cells were present in the seminiferous epithelium (Figure 4G; Table 3). In the seminiferous tubules of both operated and contralateral testes of varicocele rats treated with Se plus PDRN, very rare or no TUNEL-positive cells were observed (Figure 4H,I), so that TWAC and apoptotic index were close to sham (Table 3).

### 2.7. Administration of Se, PDRN and Their Association Modulates Caspase-1 Activity

**Figure 5:**
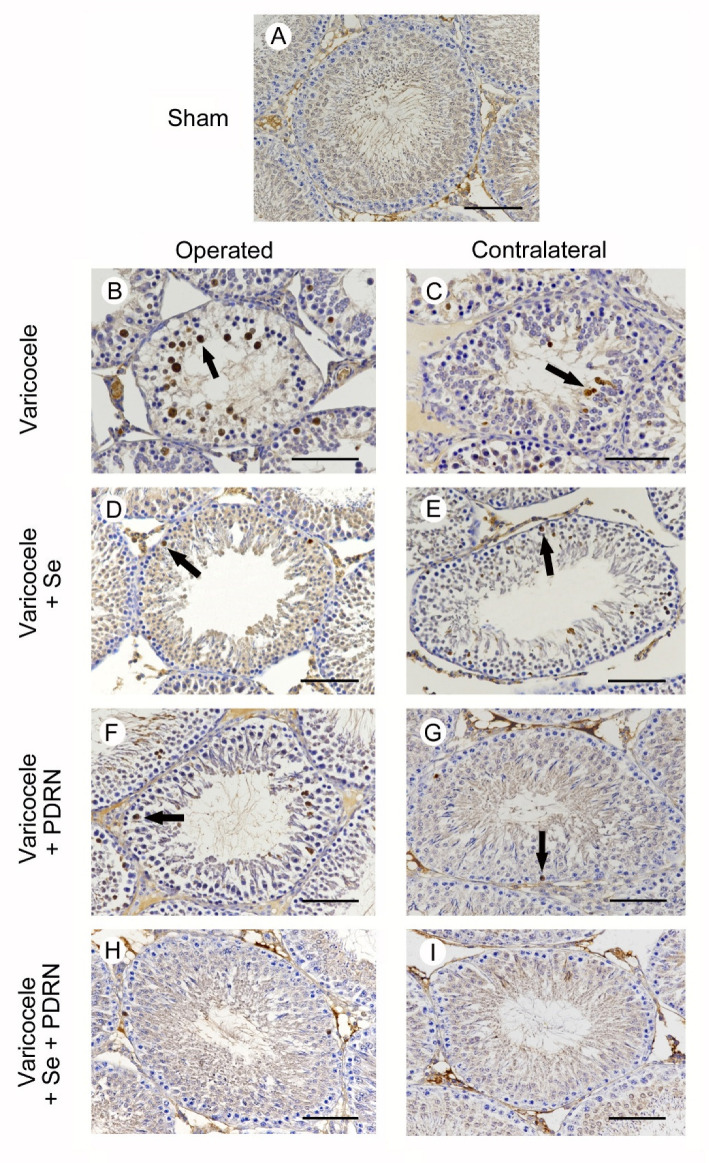
Immunohistochemical localization of caspase-1 in the testes from rats of sham, varicocele, varicocele contralateral, varicocele plus Se (0.4 mg/kg/day i.p.), varicocele plus Se contralateral, varicocele plus PDRN (8 mg/kg/day i.p.), varicocele contralateral plus PDRN, varicocele plus Se plus PDRN, varicocele contralateral plus Se plus PDRN groups. (**A**): In sham rats, no caspase-1 positive cells can be observed. (**B**): In varicocele rats, a large number of caspase-1 positive cells are located in the wall of the tubules (arrow). (**C**): In the contralateral testes of the same rats, some isolated caspase-1 positive cells are present (arrow). (**D**): In varicocele rats treated with Se, many caspase-1 positive cells are present in the periphery of the seminiferous tubules (arrow). (**E**): In the contralateral testes of the same group, some caspase-1 positive cells are located in the external part of the tubules (arrow). (**F**): In the testes of varicocele rats treated with PDRN, fewer caspase-1 positive cells are located along the periphery of the tubules (arrow). (**G**): In contralateral testes of the same group, only isolated caspase-1 positive cells (arrow) are present. (**H**,**I**): In the seminiferous tubules of both operated and contralateral testes of varicocele rats treated with Se plus PDRN, very rare or no caspase-1 positive cells are demonstrated. (Scale bar: 50 µm).

All animals of sham groups showed the same morphology when caspase-1 activity was evaluated. As a consequence, for the clarity of results, only one image is provided as representative of sham (Figure 5A) and a single datum is provided for caspase-1 positive cells (Table 3). No caspase-1 positive cells were detected in the seminiferous tubules of sham animals (Figure 5A; Table 3). In varicocele rats, a large number of caspase-1 positive cells were located in the highly damaged wall of the tubules (Figure 5B; Table 3). The number of caspase-1 positive cells was significantly reduced in the contralateral testes of the same rats (Figure 5C; Table 3). On the contrary, in varicocele rats treated with Se, many caspase-1 positive cells were present in the periphery of the seminiferous tubules (Figure 5D; Table 3). In the contralateral testes of the same group, some caspase-1 positive cells were present in the external part of the tubules (Figure 5E; Table 3). In the testes of varicocele rats treated with PDRN, a reduced number of caspase-1 positive cells were located along the periphery of the tubules (Figure 5F; Table 2). In the contralateral testes of the same group, only isolated caspase-1 positive cells were present (Figure 5G; Table 3). In the seminiferous tubules of both operated and contralateral testes of varicocele rats treated with Se plus PDRN, very rare or no caspase-1 positive cells were demonstrated (Figure 5H,I; Table 3).

### 2.8. Administration of Se, PDRN and Their Association Counteracts Ultrastructural Testes Changes

**Figure 6:**
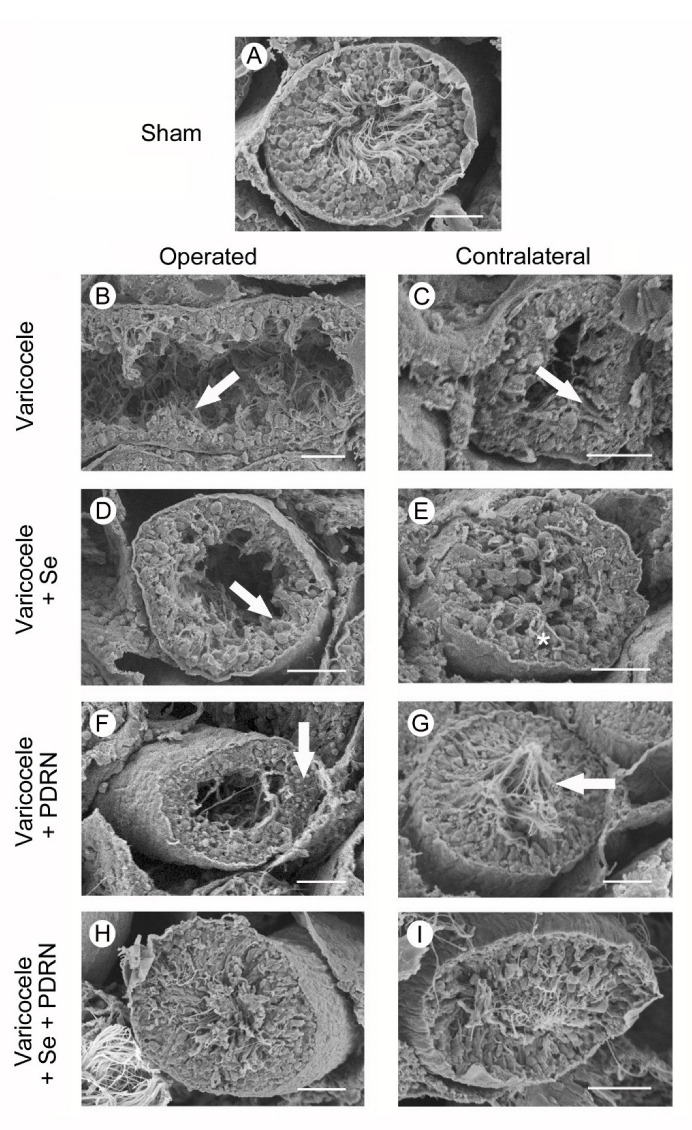
Scanning electron micrographs of testes from sham, varicocele, varicocele contralateral, varicocele plus Se (0.4 mg/kg/day i.p.), varicocele plus Se contralateral, varicocele plus PDRN (8 mg/kg/day i.p.), varicocele contralateral plus PDRN, varicocele plus Se plus PDRN, varicocele contralateral plus Se plus PDRN groups. (**A**): Sham animals. Note the normal structure of the seminiferous tubules. (**B**): Varicocele rats. Tubules show evident reduction in their height and condensed sperm tails (arrow). (**C**): In the contralateral testes of the same group, some clefts are present in the seminiferous epithelium (arrow). (**D**): In varicocele rats treated with Se, a low and irregularly arranged seminiferous epithelium is evident (arrow). (**E**): In the contralateral testes of the same group, only a few spermatozoa can be observed (asterisk). (**F**): In varicocele rats treated with PDRN the tubular lumen is reduced, owing to the presence of a higher epithelium (arrow). (**G**): In the contralateral testes of the same group, many spermatozoa are present (arrow). (**H**,**I**): In varicocele rats treated with Se plus PDRN, both the operated and the contralateral testes show close to normal organization. (Scale bar: 50 μm).

All groups of sham animals showed the same morphology as the SEM. For this reason, for the clarity of results, only one image is provided as representative of sham. In sham animals, the structure of the seminiferous tubules was normal (Figure 6A). In varicocele rats, tubules showed a reduced height of the seminiferous epithelium and condensed sperm tails (Figure 6B). The seminiferous tubules of the contralateral testes of the same group showed some clefts in the epithelium (Figure 6C). In varicocele rats treated with Se, the seminiferous epithelium was low and irregularly arranged (Figure 6D). In the contralateral testes of the same group, only few spermatozoa could be observed (Figure 6E). In the testes of varicocele rats treated with PDRN, the epithelium was higher (Figure 6F). In contralateral testes of the same group, the tubular morphology was well preserved, with evident spermatozoa (Figure 6G). In both operated and contralateral testes of varicocele rats treated with Se plus PDRN, tubules showed close to normal size and organization (Figure 6H,I).

## 3. Discussion

Infertility is an important condition affecting about 70 million couples all over the world [34]. Generally, primary and a secondary infertility are described: the first is referred to someone who has never conceived a child and is having trouble conceiving, while secondary infertility designates someone who has had one or more pregnancies in the past but is having trouble conceiving again [35].

Male infertility contributes to approximately 50%–60% of the total [1]: it is a complex multifactorial condition related to quantitative impairment of spermatogenesis, ductal obstruction or dysfunction, alteration of the hypothalamic–pituitary axis or qualitative spermatogenetic disturbances [36].

Varicocele, an abnormal dilation and/or tortuosity of the veins of the pampiniform plexus, accounts for 35% of cases, thus representing the most common cause of primary and secondary infertility [1]. Regarding the mechanism of action by which fertility is affected, a primary role is played by the increased heat induced by blood stasis in the scrotum, able to damage spermatogenesis [16]. However, metabolites reflux into the testis, increased ROS levels with sperm DNA damage, and hormonal deregulation were also considered [37]. In fact, in the present experimental model of varicocele, we were able to demonstrate the weight of both operated and contralateral testes and the level of testosterone to be significantly lower than sham, seminiferous tubules with reduced MTD and an epithelium formed only by spermatogonia with residual sperm tails. Furthermore, a large number of TUNEL-positive and caspase-1 positive cells were observed. Through Real-Time PCR, the increased mRNA expression of the NLRP3 inflammasome and of its downstream signals (caspase-1 and IL-1β) was demonstrated.

Varicocele is considered the most common cause of sub/unfertility that is correctable with surgery [1]. Microsurgical varicocelectomy is the standard operative procedure in both adolescents and adults, as it shows lower postoperative recurrence and complication rates if compared with other techniques [38,39]. Though the surgical treatment improved semen parameters and natural pregnancy rate in a significant way, further studies examining the pathophysiology of varicocele, real improvement in antioxidant defenses [30] and the involvement of new pathways are requested [1]. In this way, either the deleterious effects of varicocele on spermatogenesis could be better elucidated or new treatment strategies could be proposed in support to surgery in order to improve testicular structure, with positive effects on varicocele-induced infertility.

Among the medical approaches in support to surgery, many bioactive natural compounds are being increasingly used for therapeutics and nutraceutical applications in experimental models of varicocele. Among them, the effects of *Morinda officinalis*, resveratrol, crysin, selenium, ghrelin, silymarin, berberin, *Schisandra chinensis* and lycopene were evaluated owing to their antioxidant and anti-inflammatory activities [12,23,40,41,42,43,44,45,46]. Furthermore, our group evaluated the effects of PDRN alone and administration in experimental varicocele, demonstrating an increase in testicular angiogenesis mediated by the modulation of vascular endothelial growth factor (VEGF) -a [17] and CD-34 [18] and an inhibition of apoptosis proteins (IAPs), such as neuronal apoptosis inhibitory protein (NAIP) and survivin [33].

On the basis of the studies indicated above, in the present paper, we evaluated the role of Se and PDRN, alone or in association, on NLRP3 inflammasome activation and molecular behavior in order to provide further data in the physiopathology of varicocele to study new triggering molecular mechanisms and possibly identify potential biomarkers and/or medical targets of fertility problems.

NLRP3 inflammasome can be activated by different triggers, among which exogenous microbial stimuli, environmental large inorganic crystalline structures, and endogenous danger signals are included [47]. Given the high number of NLRP3 inflammasome activators, several studies have been performed to indicate a single molecule or pathway responsible for its activation. Three possible pathways were indicated: increased levels of ROS, a drop in intracellular potassium concentration, and the disruption of lysosomal membranes [47]. To date, oxidative stress in cells and the consequent production of ROS seems to be the major activator of the inflammasome complex [48], as recently recognized in an ischemia/reperfusion model [49], after spinal cord injury [50] and in a varicocele model [12].

When activated, NLRP3 binds to a caspase recruitment domain (CARD) and a pyrin domain. In this way, inflammasomes activate a class of caspases, a family of cysteine proteases involved in apoptosis, necrosis and inflammation, known as inflammatory caspases [47]. Their representative member, caspase-1, stimulates the secretion of cytokines such as IL-1β and IL-18 [51], which are crucial mediators of the inflammatory response.

Selenium, an essential trace element with anti-oxidative and anti-inflammatory properties, significantly reduced mRNA expression of NLRP3 inflammasome, of caspase-1 and of inflammatory cytokines in Pb-treated chicken testes and kidneys [26,52] and increased the activities of antioxidant enzymes in rats with experimental varicocele [29]. No data, however, were provided on its role on testosterone levels and apoptosis. In the present paper, Se showed positive effects on all examined parameters, but both biochemical and morphological parameters were still significantly lower if compared to sham animals.

PDRN is an adenosine A2A receptor agonist. When adenosine A2A receptors are activated, they inhibit the secretion of proinflammatory cytokines in many diseases [53,54,55,56,57,58]. Moreover, a preceding paper demonstrated that PDRN has antioxidant effects; this ROS reduction may have an indirect but significant effect on the NLRP3 inhibition [59].

The role of PDRN was already evaluated in experimental models of varicocele, demonstrating the up-regulation of the expression of VEGF-a and the inhibition of IAPs [17,18,33]. 

In this study, PDRN alone improved more significantly than Se in all considered parameters, even if none was comparable to sham operated animals.

On the contrary, the association of Se and PDRN significantly improved all the tubular morphological parameters and their ultrastructural features, increased testosterone levels, reduced NLRP3, caspase-1 and IL-1β expression and TUNEL-positive cells number, thus showing an overall positive action on fertility.

Therefore, this study suggests that NLRP3 inflammasome can be considered an interesting target for innovative bioactive compounds aimed to treat testicular injury after varicocele and that the association Se and PDRN may be used as a new medical approach in support to surgery for varicocele. Furthermore, in previous clinical trials, PDRN demonstrated a very good safety profile in patients with chronic diabetic foot ulcers and knee osteoarthritis [60,61]; thus, despite the fact that no clinical data are available regarding the efficacy of PDRN and Selenium combination to treat testicular damage, the present data suggest a potential use of this association in patients with varicocele.

Of course, additional and translational studies are required to study a new possible mechanism of action of this bioactive association in the context of healthy diet, rich in anti-inflammatory and antioxidant food, such as a Mediterranean-style diet.

Finally, these findings suggest that the association Se and PDRN may also offer a structural model for the design of new analog compounds (i.e., functional and/or medical foods) that can provide novel therapeutic approaches in the management of varicocele and male infertility.

## 4. Material and Methods

### 4.1. Experimental Protocol

All procedures were authorized by the Italian Ministry of Health (02 February 2017) (authorization number 90/2017—PR) and the Animal Research: Reporting In Vivo Experiments (ARRIVE) guidelines were followed. Male Sprague-Dawley rats (72 animals, age 7 weeks, weight 200–230 g) were provided by Charles River Laboratories Italia srl (Calco, Italy). The rats were kept under a 12-hour light/dark cycle, a temperature of about 23 °C with water and food ad libitum. A total of 36 animals underwent varicocele as previously described [18,23,33]. The other animals (*n* = 36) were sham operated. Twenty-eight days after both surgical procedures, all animals were daily administered with vehicle (*n* = 18), with seleno-L-methionine (Se), (*n* = 18; 0.4 mg/kg i.p.), with PDRN (*n* = 18; 8 mg/Kg/day i.p.), and with Se (0.4 mg/kg i.p., daily) and PDRN (8 mg/Kg/day i.p.) (*n* = 18) throughout a further experimental period of 30 days. The basis for using the said doses of Se and PDRN was specified in previous works by our group [17,18,27] and other authors [29]. All rats were then sacrificed with an intraperitoneal (i.p.) overdose of ketamine and xylazine. Both testes were weighted and analyzed for biochemical, histopathological, immunohistochemical and ultrastructural evaluation.

### 4.2. Determination of Testosterone

Testosterone determination was performed in serum by ELISA methodology using a commercially available kit, according to the manufacturer’s protocol, as previously described [23].

### 4.3. Analysis of Cytokine Expressions through Real-Time PCR

The mRNA expression of NLRP3, IL-1β and Caspase-1 was evaluated as previously described [62,63,64]. Primers used for target and reference genes were listed in Table 4.

### 4.4. Evaluation of NLRP3 and IL-1β Levels in Testis

Testis tissues were assayed using commercially available ELISA kits for NLRP3 and IL-1β (Abcam, Cambridge, UK) following the protocol of the manufacturer, as previously described [65,66].

### 4.5. Determination of Glutathion (GSH) and Glutathion Peroxidase (GPx)

GSH content and GPx activity were measured according to the manufacturer’s protocol of commercial kits (Abcam, Cambridge, UK). All the samples were evaluated in duplicate and the obtained results were interpolated with the pertinent standard curves.

### 4.6. Histological Evaluation

The testes were processed for light microscopy and photographed according to the techniques previously described in detail [23].

### 4.7. Evaluation of Apoptosis with Terminal Deoxynucleotidyl Transferase dUTP Nick End Labeling (TUNEL) Assay

An apoptosis detection kit (in situ Apoptosis Detection kit, Abcam, Cambridge, UK) was used for the TUNEL technique, following the manufacturer’s instructions. In brief, histological sections (5 µm) were permeabilized, treated with H_2_O_2_ to block endogenous peroxidase, and then incubated as previously described in detail [23]. Counterstaining was performed in Mayer’s haematoxylin. The slides were photographed with a Nikon Ci-L light microscope using a digital camera Nikon Ds-Ri2. For evaluation of the distribution of the apoptotic cells, see below (Section 4.9).

### 4.8. Immunohistochemistry for Caspase-1

Histological sections (5 µm) were treated with pH 6.0 citrate buffer and then with H_2_O_2_ to block endogenous peroxidase. Primary antibody (caspase-1, 1:250, Cell Signalling, Tucson, AZ, USA) was incubated overnight at 4 °C in a moisturized chamber and the day after the secondary antibody (anti-mouse, Vectastain, Vector, Burlingame, CA, USA) was added and the reaction was visualized with 3,3′-Diaminobenzidine (DAB) (Sigma-Aldrich, Milan, Italy). Counterstaining was performed in Mayer’s haematoxylin [67]. Appropriate positive and negative controls were used in each test. Slides were photographed with a Nikon Ci-L light microscope using a digital camera Nikon Ds-Ri2. For parameters evaluated on caspase-1 positive cells, see below (Section 4.9).

### 4.9. Morphometric Evaluation

From the specimens stained with HE, the mean tubule diameter (MTD) was calculated as previously described in detail [23] Furthermore, spermatogenesis was evaluated using the Johnsen’s scoring system (JS) [68], as modified by Erdemir et al. [69], giving a score of 10 to 1 on the basis of the epithelial structure of each considered tubule.

In order to evaluate the distribution of apoptosis, 100 seminiferous tubules of each group were considered to establish the percentage of tubules with apoptotic cells (%TWAC) and the mean number of TUNEL-positive cells per tubule, indicated as apoptotic index [70]. For the assessment of the immunoreactivity for caspase-1, the number of positive cells was counted from 10 nonserial sections of each group of rats, selecting two unit areas (UA) of 0.1 μm^2^ (316 × 316 μm).

### 4.10. Scanning Electron Microscopy

Testes from two rats of each of the above-indicated groups were fixed in 2.5% glutaraldehyde in 0.2 M phosphate buffer (pH 7.4) at +4 °C, washed with 0.2 M phosphate buffer (pH 7.4), dehydrated in graded ethanol, and then critical-point-dried in CO_2_. The testes were gently fractured, so that the inner surface of the tubules was exposed. The specimens were covered with gold and viewed and photographed in a JEOL JCM 6000 (JEOL, Tokyo, Japan) scanning electron microscope adjusted at 15 kV.

### 4.11. Drugs

Mastelli Srl, Sanremo, Italy, kindly provided PDRN. All chemicals and reagents were commercially available reagent grades.

### 4.12. Statistical Analysis

A post-hoc analysis between any two experimental groups was handled with the Student’s *t*-test using the Bonferroni correction to account for multiple comparisons. A *p* value of ≤ 0.05 was considered statistically significant. Values are provided as mean ± standard error (SE).

## Figures and Tables

**Table 1:** Effects on testis weight, testosterone, mean tubular diameter (MTD) and Johnsen’s score (JS) induced by Se (0.4 mg/kg i.p.), PDRN (8 mg/Kg/day i.p.) and Se (0.4 mg/kg i.p.) plus PDRN (8 mg/Kg/ day i.p.) in varicocele rats as compared to sham and varicocele rats. Se = seleno-L-methionine; PDRN = polydeoxyribonucleotide; CL = contralateral testis. All values are expressed as mean ± SE; *n* = 7 animals for each group. ^a^ *p* < 0.05 vs. sham; ^b^ *p* < 0.05 vs. varicocele.

Groups	Testis Weight (g)	Testosterone (ng/ml)	MTD (μm)	JS
Sham	1.65 ± 0.17	6.1 ± 0.9	249 ± 21	9.7 ± 0.2
Varicocele	0.83 ± 0.11 ^a^	2.6 ± 0.5 ^a^	127 ± 19 ^a^	2.6 ± 0.5 ^a^
Varicocele CL	1.12 ± 0.36 ^a,b^		168 ± 15 ^a,b^	7.3 ± 0.8 ^a,b^
Varicocele + Se	1.18 ± 0.25 ^a,b^	4.2 ± 0.8 ^a,b^	173 ± 12 ^a,b^	6.7 ± 0.7 ^a,b^
Varicocele + Se CL	1.39 ± 0.33 ^a,b^		203 ± 16 ^a,b^	8.8 ± 0.6 ^b^
Varicocele + PDRN	1.28 ± 0.10 ^a,b^	5.1 ± 0.5 ^b^	210 ± 15 ^a,b^	8.4 ± 1.3 ^b^
Varicocele + PDRN CL	1.55 ± 0.51 ^b^		221 ± 13 ^b^	9.1 ± 0.7 ^b^
Varicocele + PDRN + Se	1.58 ± 0.35 ^b^	5.9 ± 0.7 ^b^	242 ± 17 ^b^	9.0 ± 0.5 ^b^
Varicocele + PDRN + Se CL	1.62 ± 0.39 ^b^		251 ± 14 ^b^	9.3 ± 0.5 ^b^

**Table 2:** Effects on glutathione (GSH) and glutathione peroxidase (GPx) as induced by Se (0.4 mg/kg i.p.), PDRN (8 mg/Kg/day i.p.) and Se (0.4 mg/kg i.p.) plus PDRN (8 mg/Kg/ day i.p.) in varicocele rats as compared to sham and varicocele rats. Se = seleno-L-methionine; PDRN = polydeoxyribonucleotide; CL = contralateral testis. All values are expressed as mean± SE; *n* = 7 animals for each group. ^a^ *p* < 0.05 vs. sham; ^b^ *p* < 0.05 vs. varicocele.

Groups	GSH (nmol/mg Tissue)	GPx (nmol/min/mg Tissue)
Sham	46 ± 3	52 ± 4
Varicocele	13 ± 2 ^a^	26 ± 2 ^a^
Varicocele + CL	25 ± 2 ^a,b^	37 ± 6 ^a,b^
Varicocele + Se	24 ± 3 ^a,b^	34 ± 5 ^a,b^
Varicocele + Se CL	27 ± 2 ^a,b^	44 ± 4 ^a,b^
Varicocele + PDRN	33 ± 2 ^a,b^	43 ± 5 ^a,b^
Varicocele + PDRN CL	37 ± 3 ^b^	49 ± 6 ^b^
Varicocele + PDRN + Se	42 ± 3 ^b^	47 ± 7 ^b^
Varicocele + PDRN + Se CL	45 ± 3 ^b^	50 ± 4 ^b^

**Table 3:** Effects on %TWAC (percentage of tubules with apoptotic cells), on the apoptotic index (mean number of TUNEL-positive cells per tubule), and on the number of caspase-1 positive cells/MF (microscopic field) as induced by Se (0.4 mg/kg i.p.), PDRN (8 mg/Kg/day i.p.) and Se (0.4 mg/kg i.p.) plus PDRN (8 mg/Kg/ day i.p.) in varicocele rats as compared to sham and varicocele rats. Se = seleno-L-methionine; PDRN = polydeoxyribonucleotide; CL = contralateral testis. All values are expressed as mean ± SE; *n* = 7 animals for each group. ^a^ *p* < 0.05 vs. sham; ^b^ *p* < 0.05 vs. varicocele.

Groups	% TWAC	Apoptotic Index	Caspase-1 Positive Cells/MF
Sham	0.2 ± 0.1	0.1 ± 0.1	0.3 ± 0.1
Varicocele	35 ± 5 ^a^	10 ± 2.6 ^a^	15.2 ± 4.1 ^a^
Varicocele + CL	7.5 ± 2.6 ^a,b^	4.1 ± 1.4 ^a,b^	7.5 ± 2.1 ^a,b^
Varicocele + Se	13.2 ± 2.7 ^a,b^	7.1 ± 1.4 ^a,b^	6.3 ± 1.7 ^a,b^
Varicocele + Se CL	3.3 ± 1.3 ^a,b^	3.9 ± 1.2 ^a,b^	3.5 ± 1.3 ^a,b^
Varicocele + PDRN	7.2 ± 1.7 ^a,b^	5.3 ± 1.1 ^a,b^	5.1 ± 1.6 ^a,b^
Varicocele + PDRN CL	1.8 ± 0.4 ^b^	2.2 ± 0.5 ^b^	2.1 ± 0.7 ^b^
Varicocele + PDRN + Se	4.4 ± 0.8 ^b^	1.5 ± 0.6 ^b^	1.3 ± 0.3 ^b^
Varicocele + PDRN + Se CL	1.3 ± 0.2 ^b^	0.2 ± 0.1 ^b^	0.4 ± 0.1 ^b^

**Table 4:** Primer list.

Gene	Sequence
β-actin	Fw:5′AGCCATGTACGTAGCCATCC3′
	Rw:5′CTCTCAGCTGTGGTGGTGAA3′
NLRP3	Fw:5′ACGGCAAGTTCGAAAAAGGC3′
	Rw:5′AGACCTCGGCAGAAGCTAGA3′
IL-1β	Fw:5′AGGCTTCCTTGTGCAAGTGT3′
	Rw:5′TGAGTGACACTGCCTTCCTG3′
Caspase-1	Fw:5′GACAAGATCCTGAGGGCAAA3′
	Rw:5′ GGTCTCGTGCCTTTTCCATA3′

## Data Availability

The datasets generated for this study are available on request to the corresponding author.

## References

[1] Cho C.L., Esteves S.C., Agarwal A. (2016). Novel insights into the pathophysiology of varicocele and its association with reactive oxygen species and sperm DNA fragmentation. Asian J. Androl..

[2] Alsaikhan B., Alrabeeah K., Delouya G., Zini A. (2016). Epidemiology of varicocele. Asian J. Androl..

[3] Hassanin A.M., Ahmed H.H., Kaddah A.N. (2018). A global view of the pathophysiology of varicocele. Andrology.

[4] Pastuszak A.W., Wang R. (2015). Varicocele and testicular function. Asian J. Androl..

[5] Barati E., Nikzad H., Karimian M. (2020). Oxidative stress and male infertility: Current knowledge of pathophysiology and role of antioxidant therapy in disease management. Cell. Mol. Life. Sci..

[6] Aitken R.J., De Iuliis G.N., Finnie J.M., Hedges A., McLachlan R. (2010). Analysis of the relationships between oxidative stress, DNA damage and sperm vitality in a patient population: Development of diagnostic criteria. Hum. Reprod..

[7] Walczak-Jedrzejowska R., Wolski J.K., Slowikowska-Hilczer J. (2013). The role of oxidative stress and antioxidants in male fertility. Cent. Eur. J. Urol..

[8] Schroder K., Tschopp J. (2010). The inflammasomes. Cell.

[9] Martinon F., Burns K., Tschopp J. (2002). The inflammasome: A molecular platform triggering activation of inflammatory caspases and processing of proIL-beta. Mol. Cell.

[10] Kayagaki N., Warming S., Lamkanfi M., Vande Walle L., Louie S., Dong J., Newton K., Qu Y., Liu J., Heldens S. (2011). Non-canonical inflammasome activation targets caspase-11. Nature.

[11] Lu Y., Xu S., Chen H., He M., Deng Y., Cao Z., Pi H., Chen C., Li M., Ma Q. (2016). CdSe/ZnS quantum dots induce hepatocyte pyroptosis and liver inflammation via NLRP3 inflammasome activation. Biomaterials.

[12] Hajipour E., Mashayekhi F.J., Mosayebi G., Baazm M., Zendedel A. (2018). Resveratrol decreases apoptosis and NLRP3 complex expressions in experimental varicocele rat model. Iran J. Basic Med. Sci..

[13] Baazm M., Ghafarizadeh A.A., Noshad Kamran A.R., Beyer C., Zendedel A. (2020). Presence of The NLRP3 Inflammasome Components in Semen of Varicocele Patients. Int. J. Fertil. Steril..

[14] Cho C.L., Esteves S.C., Agarwal A. (2019). Indications and outcomes of varicocele repair. Panminerva Med..

[15] Grasso M., Lania C., Castelli M., Galli L., Franzoso F., Rigatti P. (2000). Low-grade left varicocele in patients over 30 years old: The effect of spermatic vein ligation on fertility. B.J.U. Int..

[16] Jensen C.F.S., Østergren P., Dupree J.M., Ohl D.A., Sønksen J., Fode M. (2017). Varicocele and male infertility. Nat. Rev. Urol..

[17] Minutoli L., Arena S., Bonvissuto G., Bitto A., Polito F., Irrera N., Arena F., Fragalà E., Romeo C., Nicotina P.A. (2011). Activation of adenosine A2A receptors by polydeoxyribonucleotide increases vascular endothelial growth factor and protects against testicular damage induced by experimental varicocele in rats. Fertil. Steril..

[18] Arena S., Minutoli L., Arena F., Nicotina P.A., Romeo C., Squadrito F., Altavilla D., Morgia G., Magno C. (2012). Polydeoxyribonucleotide administration improves the intra-testicular vascularization in rat experimental varicocele. Fertil. Steril..

[19] Chen Y.W., Niu Y.H., Wang D.Q., Li H., Pokhrel G., Xu H., Wang T., Wang S.G., Liu J.H. (2018). Effect of adjuvant drug therapy after varicocelectomy on fertility outcome in males with varicocele-associated infertility: Systematic review and meta-analysis. Andrologia.

[20] Busetto G.M., Agarwal A., Virmani A., Antonini G., Ragonesi G., Del Giudice F., Micic S., Gentile V., De Berardinis E. (2018). Effect of metabolic and antioxidant supplementation on sperm parameters in oligo-astheno-teratozoospermia, with and without varicocele: A double-blind placebo-controlled study. Andrologia.

[21] Kefer J.C., Agarwal A., Sabanegh E. (2009). Role of antioxidants in the treatment of male infertility. Int. J. Urol..

[22] Razi M., Tavalaee M., Sarrafzadeh-Rezaei F., Moazamian A., Gharagozloo P., Drevet J.R., Nasr-Eshafani M.H. (2020). Varicocele and Oxidative Stress: New Perspectives from Animal and Human Studies. Andrology.

[23] Antonuccio P., Micali A., Puzzolo D., Romeo C., Vermiglio G., Squadrito V., Freni J., Pallio G., Trichilo V., Righi M. (2020). Nutraceutical Effects of Lycopene in Experimental Varicocele: An “In Vivo” Model to Study Male Infertility. Nutrients.

[24] Minutoli L., Marini H., Aomori C., Hokkaido M. (2012). Selenium and prostate health: A new possible nutraceutical challenge. Selenium sources, functions and health effects. Public Health in the 21st Century, Nutrition and Diet Research Progressed.

[25] Minutoli L., Squadrito F., Altavilla D., Marini H., Preedy V.R. (2015). Therapy with Selenium Cocktails and Co-use of Lycopene and Selenium. Selenium: Chemistry, Analysis, Function and Effects (Food and Nutritional Components in Focus).

[26] Huang H., Jiao X.Y., Xu Y.M., Han Q., Jiao W.Y., Liu Y.Y., Li S., Teng X. (2019). Dietary selenium supplementation alleviates immune toxicity in the hearts of chickens with lead-added drinking water. Avian Pathol..

[27] Benvenga S., Micali A., Pallio G., Vita R., Malta C., Puzzolo D., Irrera N., Squadrito F., Altavilla D., Minutoli L. (2019). Effects of Myo-inositol Alone and in Combination with Seleno-L-methionine on Cadmium-Induced Testicular Damage in Mice. Curr. Mol. Pharmacol..

[28] Huang H., Li X., Wang Z., Lin X., Tian Y., Zhao Q., Zheng P. (2020). Anti-inflammatory effect of selenium on lead-induced testicular inflammation by inhibiting NLRP3 inflammasome activation in chickens. Theriogenology.

[29] Taghizadeh L., Eidi A., Mortazavi P., Rohani A.H. (2017). Effect of selenium on testicular damage induced by varicocele in adult male Wistar rats. J. Trace Elem. Med. Biol..

[30] Ardestani Zadeh A., Arab D., Kia N.S., Heshmati S., Amirkhalili S.N. (2019). The role of Vitamin E—Selenium—Folic Acid Supplementation in Improving Sperm Parameters After Varicocelectomy: A Randomized Clinical Trial. Urol. J..

[31] Altavilla D., Bitto A., Polito F., Marini H., Minutoli L., Di Stefano V., Irrera N., Cattarini G., Squadrito F. (2009). Polydeoxyribonucleotide (PDRN): A safe approach to induce therapeutic angiogenesis in peripheral artery occlusive disease and in diabetic foot ulcers. Cardiovasc. Hematol. Agents Med. Chem..

[32] Squadrito F., Micali A., Rinaldi M., Irrera N., Marini H., Puzzolo D., Pisani A., Lorenzini C., Valenti A., Laurà R. (2017). Polydeoxyribonucleotide, an Adenosine-A2A Receptor Agonist, Preserves Blood Testis Barrier from Cadmium-Induced Injury. Front. Pharmacol..

[33] Minutoli L., Arena S., Antonuccio P., Romeo C., Bitto A., Magno C., Rinaldi M., Micali A., Irrera N., Pizzino G. (2015). Role of Inhibitors of Apoptosis Proteins in Testicular Function and Male Fertility: Effects of Polydeoxyribonucleotide Administration in Experimental Varicocele. Biomed. Res. Int..

[34] Boivin J., Bunting L., Collins J.A., Nygren K.G. (2007). International estimates of infertility prevalence and treatment-seeking: Potential need and demand for infertility medical care. Hum. Reprod..

[35] Leaver R.B. (2016). Male infertility: An overview of causes and treatment options. Br. J. Nurs..

[36] Krausz C., Riera-Escamilla A. (2018). Genetics of male infertility. Nat. Rev. Urol..

[37] Fainberg J., Kashanian J.A. (2019). Recent advances in understanding and managing male infertility. F1000Res..

[38] Al-Said S., Al-Naimi A., Al-Ansari A., Younis N., Shamsodini A., A-sadiq K., Shokeir A.A. (2008). Varicocelectomy for male infertility: A comparative study of open, laparoscopic and microsurgical approaches. J. Urol..

[39] Mohamed E.E., Gawish M., Mohamed A. (2017). Semen parameters and pregnancy rates after microsurgical varicocelectomy in primary versus secondary infertile men. Hum Fertil..

[40] Zhang L., Zhao X., Wang F., Lin Q., Wang W. (2016). Effects of Morinda officinalis Polysaccharide on Experimental Varicocele Rats. Evid. Based Complement Alternat. Med..

[41] Mendes T.B., Paccola C.C., De Oliveira Neves F.M., Simas J.N., Da Costa Vaz A., Cabral R.E., Vendramini V., Miraglia S.M. (2016). Resveratrol improves reproductive parameters of adult rats varicocelized in peripuberty. Reproduction.

[42] Missassi G., Dos Santos Borges C., De Lima Rosa J., Villela E., Silva P., Da Cunha Martins A., Barbosa F., De Grava Kempinas W. (2017). Chrysin Administration Protects against Oxidative Damage in Varicocele-Induced Adult Rats. Oxid. Med. Cell. Longev..

[43] Asadi N., Kheradmand A., Gholami M., Moradi F.H. (2018). Effect of ghrelin on the biochemical and histopathology parameters and spermatogenesis cycle following experimental varicocele in rat. Andrologia.

[44] Mazhari S., Razi M., Sadrkhanlou R. (2018). Silymarin and celecoxib ameliorate experimental varicocele-induced pathogenesis: Evidences for oxidative stress and inflammation inhibition. Int. Urol. Nephrol..

[45] Hassani-Bafrani H., Najaran H., Razi M., Rashtbari H. (2019). Berberine ameliorates experimental varicocele-induced damages at testis and sperm levels; evidences for oxidative stress and inflammation. Andrologia.

[46] Karna K.K., Choi B.R., Kim M.J., Kim H.K., Park J.K. (2019). The Effect of Schisandra chinensis Baillon on Cross-Talk between Oxidative Stress, Endoplasmic Reticulum Stress, and Mitochondrial Signaling Pathway in Testes of Varicocele-Induced SD Rat. Int. J. Mol. Sci..

[47] Abderrazak A., Syrovets T., Couchie D., El Hadri K., Friguet B., Simmet T., Rouis M. (2015). NLRP3 inflammasome: From a danger signal sensor to a regulatory node of oxidative stress and inflammatory diseases. Redox Biol..

[48] Tschopp J., Schroder K. (2010). NLRP3 inflammasome activation: The convergence of multiple signalling pathways on ROS production?. Nat. Rev. Immunol..

[49] Minutoli L., Antonuccio P., Irrera N., Rinaldi M., Bitto A., Marini H., Pizzino G., Romeo C., Pisani A., Santoro G. (2015). NLRP3 Inflammasome Involvement in the Organ Damage and Impaired Spermatogenesis Induced by Testicular Ischemia and Reperfusion in Mice. J. Pharmacol. Exp. Ther..

[50] Bazrafkan M., Nikmehr B., Shahverdi A., Hosseini S.R., Hassani F., Poorhassan M., Mokhtari T., Abolhassani F., Choobineh H., Beyer C. (2018). Lipid Peroxidation and Its Role in the Expression of NLRP1a and NLRP3 Genes in Testicular Tissue of Male Rats: A Model of Spinal Cord Injury. Iran Biomed. J..

[51] Khodamoradi K., Amini-Khoei H., Khosravizadeh Z., Hosseini S.R., Dehpour A.R., Hassanzadeh G. (2019). Oxidative stress, inflammatory reactions and apoptosis mediated the negative effect of chronic stress induced by maternal separation on the reproductive system in male mice. Reprod. Biol..

[52] Huang H., Chen J., Sun Q., Liu Y., Tang Y., Teng X. (2020). NLRP3 inflammasome is involved in the mechanism of mitigative effect of selenium on lead-induced inflammatory damage in chicken kidneys. Environ. Sci. Pollut. Res. Int..

[53] Rho J.H., Ko I.G., Jin J.J., Hwang L., Kim S.H., Chung J.Y., Hwang T.J., Han J.H. (2020). Polydeoxyribonucleotide Ameliorates Inflammation and Apoptosis in Achilles Tendon-Injury Rats. Int. Neurourol. J..

[54] Kim S.E., Ko I.G., Jin J.J., Hwang L., Kim C.J., Kim S.H., Han J.H., Jeon J.W. (2020). Polydeoxyribonucleotide Exerts Therapeutic Effect by Increasing VEGF and Inhibiting Inflammatory Cytokines in Ischemic Colitis Rats. Biomed. Res. Int..

[55] Irrera N., Bitto A., Vaccaro M., Mannino F., Squadrito V., Pallio G., Arcoraci V., Minutoli L., Ieni A., Lentini M. (2020). PDRN, a Bioactive Natural Compound, Ameliorates Imiquimod-Induced Psoriasis through NF-κB Pathway Inhibition and Wnt/β-Catenin Signaling Modulation. Int. J. Mol. Sci..

[56] Marini H.R., Puzzolo D., Micali A., Adamo E.B., Irrera N., Pisani A., Pallio G., Trichilo V., Malta C., Bitto A. (2018). Neuroprotective Effects of Polydeoxyribonucleotide in a Murine Model of Cadmium Toxicity. Oxid. Med. Cell. Longev..

[57] Pizzino G., Irrera N., Galfo F., Oteri G., Atteritano M., Pallio G., Mannino F., D’Amore A., Pellegrino E., Aliquò F. (2017). Adenosine Receptor Stimulation Improves Glucocorticoid-Induced Osteoporosis in a Rat Model. Front. Pharmacol..

[58] Pallio G., Bitto A., Pizzino G., Galfo F., Irrera N., Squadrito F., Squadrito G., Pallio S., Anastasi G.P., Cutroneo G. (2016). Adenosine Receptor Stimulation by Polydeoxyribonucleotide Improves Tissue Repair and Symptomology in Experimental Colitis. Front. Pharmacol..

[59] Kim Y.J., Kim M.J., Kweon D.K., Lim S.T., Lee S.J. (2020). Polydeoxyribonucleotide Activates Mitochondrial Biogenesis but Reduces MMP-1 Activity and Melanin Biosynthesis in Cultured Skin Cells. Appl. Biochem. Biotechnol..

[60] Squadrito F., Bitto A., Altavilla D., Arcoraci V., De Caridi G., De Feo M.E., Corrao S., Pallio G., Sterrantino C., Minutoli L. (2014). The effect of PDRN, an adenosine receptor A2A agonist, on the healing of chronic diabetic foot ulcers: Results of a clinical trial. J. Clin. Endocrinol. Metab..

[61] Kim M.S., Cho R.K., In Y. (2019). The efficacy and safety of polydeoxyribonucleotide for the treatment of knee osteoarthritis: Systematic review and meta-analysis of randomized controlled trials. Medicine.

[62] Gugliandolo E., D’Amico R., Cordaro M., Fusco R., Siracusa R., Crupi R., Impellizzeri D., Cuzzocrea S., Di Paola R. (2018). Neuroprotective Effect of Artesunate in Experimental Model of Traumatic Brain Injury. Front. Neurol..

[63] Irrera N., D’Ascola A., Pallio G., Bitto A., Mannino F., Arcoraci V., Rottura M., Ieni A., Minutoli L., Metro D. (2020). β-Caryophyllene Inhibits Cell Proliferation through a Direct Modulation of CB2 Receptors in Glioblastoma Cells. Cancers.

[64] Irrera N., Arcoraci V., Mannino F., Vermiglio G., Pallio G., Minutoli L., Bagnato G., Anastasi G.P., Mazzon E., Bramanti P. (2018). Activation of A2A Receptor by PDRN Reduces Neuronal Damage and Stimulates WNT/β-CATENIN Driven Neurogenesis in Spinal Cord Injury. Front. Pharmacol..

[65] Irrera N., D’Ascola A., Pallio G., Bitto A., Mazzon E., Mannino F., Squadrito V., Arcoraci V., Minutoli L., Campo G.M. (2019). β-Caryophyllene Mitigates Collagen Antibody Induced Arthritis (CAIA) in Mice Through a Cross-Talk between CB2 and PPAR-γ Receptors. Biomolecules.

[66] Pallio G., Micali A., Benvenga S., Antonelli A., Marini H.R., Puzzolo D., Macaione V., Trichilo V., Santoro G., Irrera N. (2019). Myo-inositol in the protection from cadmium-induced toxicity in mice kidney: An emerging nutraceutical challenge. Food Chem. Toxicol..

[67] Pallio G., Bitto A., Ieni A., Irrera N., Mannino F., Pallio S., Altavilla D., Squadrito F., Scarpignato C., Minutoli L. (2020). Combined Treatment with Polynucleotides and Hyaluronic Acid Improves Tissue Repair in Experimental Colitis. Biomedicines.

[68] Johnsen S.G. (1970). Testicular biopsy score count—A method for registration of spermatogenesis in human testes: Normal values and results in 335 hypogonadal males. Hormones.

[69] Erdemir F., Atilgan D., Markoc F., Boztepe O., Suha-Parlaktas B., Sahin S. (2012). The effect of diet induced obesity on testicular tissue and serum oxidative stress parameters. Actas Urol. Esp..

[70] Tsounapi P., Saito M., Dimitriadis F., Kitatani K., Kinoshita Y., Shomori K., Takenaka A., Satoh K. (2012). The role of K ATP channels on ischemia-reperfusion injury in the rat testis. Life Sci..

